# Comparison of two immunohistochemical staining protocols for ALK demonstrates non-inferiority of a 5A4 clone-based protocol versus an ALK01 clone-based protocol for the diagnosis of ALK + anaplastic large cell lymphoma

**DOI:** 10.1007/s12308-023-00531-0

**Published:** 2023-01-31

**Authors:** Sebastian Fernandez-Pol, Cristiane R. Ferreira, Vidhya Manohar, José Antonio Sanches, Luis A. P. C. Lage, Juliana Pereira, Maria C. N. Zerbini, Dita Gratzinger, Yasodha Natkunam

**Affiliations:** 1 Department of Pathology, Stanford University School of Medicine, 300 Pasteur Drive, Stanford, CA 94305 USA; 2 Department of Pathology, University of Sao Paulo, Sao Paulo, SP 01246 Brazil; 3 Department of Dermatology, University of Sao Paulo, Sao Paulo, SP 01246 Brazil; 4 Department of Hematology, University of Sao Paulo, Sao Paulo, SP 01246 Brazil

**Keywords:** ALK, Anaplastic large cell lymphoma, Immunohistochemistry

## Abstract

Detection of ALK rearrangement and/or expression of the ALK protein is an essential component in the evaluation of many neoplasms. Variability has been reported in the ability of different antibody clones to detect ALK expression. The ALK01 clone is commonly used to detect ALK expression in ALK-positive anaplastic large cell lymphoma (ALK + ALCL). However, this clone has been shown to lack sensitivity when used for solid tumors. The aim of this study was to determine if our high-sensitivity 5A4-based immunohistochemistry protocol is non-inferior to our ALK01-based protocol for the detection of ALK expression in ALK + ALCL. To compare the two protocols, we stained tissue microarrays of 126 hematolymphoid neoplasms and an additional 21 primary cutaneous ALK-negative anaplastic large cell lymphomas with both protocols. All 28 ALK + ALCL samples that were positive for the ALK01 antibody were also positive for the 5A4 clone. Three cases on the tissue microarray that were negative with the ALK01 antibody were clearly positive with the 5A4 antibody. We subsequently stained whole tissue sections of these three cases with the ALK01 antibody and found that these three cases were indeed positive with the ALK01 protocol, suggesting that the absence of staining on the tissue microarray samples was due to a combination of sampling error as well as a dimmer signal with the ALK01 protocol. Our study demonstrates that our 5A4-based protocol is non-inferior to the ALK01 antibody for the diagnosis of ALK-positive anaplastic large cell lymphoma, thus allowing our laboratory to discontinue the use of the ALK01-based protocol.

## Introduction

The *ALK1* oncogene plays a critical role in the pathogenesis of a wide variety of both hematolymphoid and non-hematolymphoid neoplasms. Many aberrations involving the *ALK1* oncogene are rearrangements that lead to the fusion of the catalytic tyrosine kinase domain of *ALK1* to a partner that leads to inappropriate expression of the ALK protein, which is normally only expressed in a subpopulation of cells in the developing embryonic and neonatal brain [1]. The detection of *ALK1* rearrangements or expression of the *ALK1* protein has become the standard of care to diagnose a variety of tumors and to predict responsiveness to therapies that target the ALK tyrosine kinase. Among anaplastic large cell lymphomas (ALCLs), the separation of ALK-positive from ALK-negative tumors is required because the outcome of ALK-positive ALCL is generally superior to that of most ALK-negative ALCL subtypes [2–5]. ALK expression is also useful to distinguish primary cutaneous ALCL, which is ALK-negative, from cutaneous involvement by a systemic ALK-positive ALCL. Therefore, a sensitive immunodiagnostic assay to detect ALK protein is of high clinical relevance.

In order to detect diagnostically and therapeutically relevant ALK abnormalities, many centers, including ours, perform immunohistochemical and/or cytogenetic studies. Detection of *ALK1* expression by immunohistochemistry has been shown to be a reliable surrogate for *ALK1* rearrangements and thus represents a useful tool in the routine diagnostic classification of tumors. Numerous *ALK1* antibodies are commercially available, and several of them have been compared in prior studies, with some variability in sensitivity and specificity [6–11]. Our clinical immunodiagnostic lab has used an immunohistochemical stain protocol based on the ALK01 clone to detect ALK expression in hematolymphoid tumors. Based on a discussion with laboratory directors at other institutions, the ALK01 clone has been a commonly used antibody for the detection of ALK expression in ALK-positive anaplastic large-cell lymphoma. Though the ALK01 clone is the predominant clone used for the detection of ALK expression in ALK-positive anaplastic large cell lymphoma, it is well documented that it is less sensitive than other protocols for the detection of ALK expression in non-hematolymphoid tumors such as non-small cell lung cancer [7]. This has led some laboratories to validate and maintain a separate ALK stain using a high-sensitivity ALK protocol for use in solid tumors in addition to ALK01-based protocols for ALK-positive anaplastic large cell lymphoma. In our laboratory, in addition to the ALK01 protocol, we validated a high-sensitivity ALK assay using the 5A4 antibody to detect ALK expression in non-hematolymphoid neoplasms. This high-sensitivity ALK assay was validated and has thus far passed all College of American Pathologists high-sensitivity ALK assay proficiency testing surveys between 2019 and 2022. In this study, we set out to determine if the 5A4-based protocol used in our laboratory is non-inferior to the ALK01-based protocol for the diagnosis of ALK-positive ALCL. The results of this study demonstrate that our 5A4-based protocol is non-inferior to the ALK01-based protocol, thus providing the rationale for the discontinuation of our ALK01-based protocol.

## Materials and methods

### Immunohistochemistry

The ALK01 antibody was obtained from Dako (Dako, Carpinteria, CA, catalog # M7195) and used at a dilution of 1:75 with the Ventana proprietary CC1 antigen retrieval solution, pH 8.5, on a Ventana XT instrument (Ventana Medical Systems, Tucson, AZ). The 5A4 clone was obtained from Abcam (Abcam, Cambridge, MA, USA, catalog # ab17127) and used at a dilution of 1:25 with the Leica proprietary ER2 antigen retrieval solution on a Leica BOND-III instrument (Leica Biosystems, Newcastle Upon Tyne, UK). This antibody protocol was validated for the detection of ALK-rearranged lung adenocarcinomas and inflammatory myofibroblastic tumors. This stain has routinely demonstrated expected results in College of American Pathologists proficiency testing over several years with the following results: 2019—9 of 9 correct results, 2020—19 of 19 correct results, 2021—17 of 17 correct results.

### Case selection

Cases for the tissue microarrays (TMAs) were obtained from the Archives of the Division of Anatomic Pathology, the Sao Paulo University Faculty of Medicine, and the Department of Pathology at Stanford University Medical Center. Institutional review board approval was obtained from both institutions. Representative duplicate cores of 0.6 mm were used for tissue microarray construction after careful selection based on H&E and CD30-stained sections, as previously described [12, 13]. In total, the TMAs initially included 123 individual cases. After staining, 102 individual cases with sufficient interpretable tissue in both ALK01- and 5A4-stained TMAs remained. The final analysis included 28 ALK-positive ALCL, 37 ALK-negative ALCL, and several categories of other lymphomas in the differential diagnosis of ALCL, as listed in Table 1. Four of the ALK − ALCLs harbored the *DUSP22/IRF4* translocation, and none harbored the *TP63* translocations. Non-hematopoietic tissue controls (12) were also included. We also stained whole sections of 21 cases of primary cutaneous ALK-negative anaplastic large cell lymphomas that were obtained from the archives of the Dermatopathology Section from the Department of Dermatology at the University of Sao Paulo Medical School.

**Table 1:** Summary of cases included in the final analysis

Final diagnosis	Source (TMA or whole sections)	Number of cases	Number positive with ALK01	Number positive with 5A4	Comments
ALK + ALCL	TMA (*n* = 31) Whole sections (*n* = 3)	31	31 (including 3 initially called negative on TMA then positive on whole sections)	31	Includes cases 3, 4, and 5 that were reclassified from ALK- ALCL after staining whole sections
ALK − ALCL	TMA (*n* = 34)	34	0	0	
Primary cutaneous ALK − ALCL	Whole sections (*n* = 21)	21	0	0	
PTCL, NOS	TMA (*n* = 19)	19	0	0	
AITL	TMA (*n* = 1)	1	0	0	
ENKTL	TMA (*n* = 7)	7	0	0	
CHL	TMA (*n* = 10)	10	0	0	
Total		123	31	31	

*TMA*, tissue microarray; *ALK − ALCL*, ALK-negative anaplastic large cell lymphoma; *PTCL*, peripheral T cell lymphoma, not otherwise specified; *CHL*, classic Hodgkin lymphoma; *NK/T NHL*, NK/T cell lymphoma; *AITL*, angioimmunoblastic T cell lymphoma

## Results and discussion

**Fig. 1:**
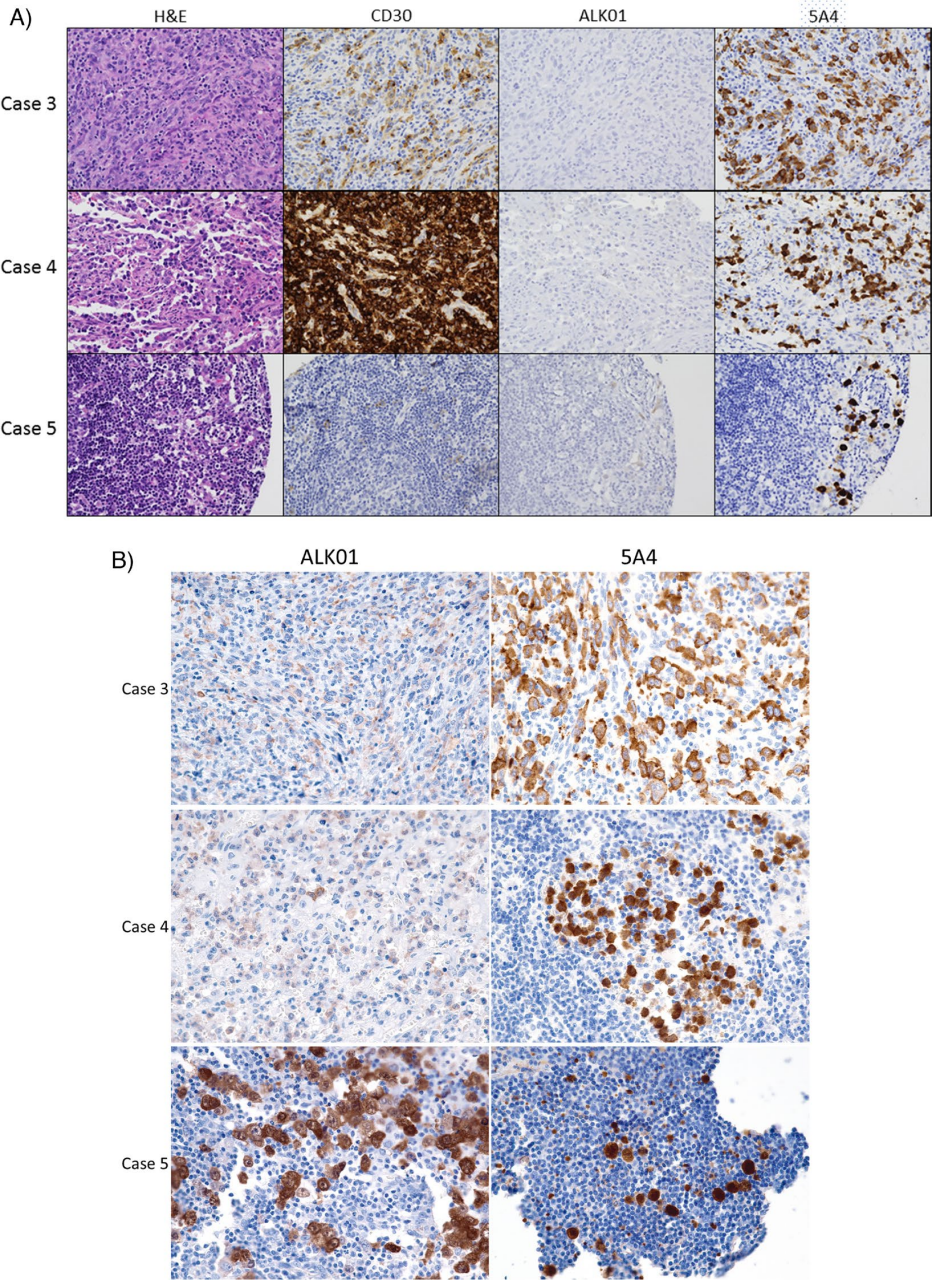
Cases that were classified as ALK-negative ALCL originally. **A** The images show tissue from the tissue microarrays of three cases that were initially classified as ALK-negative ALCL on the basis of a negative ALK01 stain but showed strong positivity with the 5A4 antibody. Rare dimly positive cells that could be misinterpreted as nonspecific staining were seen in the ALK01-stained samples. Case 5 showed rare dim CD30-positive cells and very rare dimly ALK01-positive cells. 5A4 brightly highlights rare cells. Images are × 400 magnification. **B** Whole tissue sections of cases 3, 4, and 5 were stained with the ALK01 and 5A4 protocols. In cases 3 and 4, the ALK01 protocol revealed ALK expression in the lymphoma cells, but the signal was notably dimmer than with the 5A4 protocol

As expected, given that diagnostic classification of these cases was performed using the ALK01 antibody protocol, all 28 ALK-positive ALCL cases with interpretable tissue on the TMAs were positive when stained with the ALK01 protocol. The remaining 95 cases stained were negative for ALK expression with the ALK01 protocol. Interestingly, three cases that had been initially classified as ALK-negative ALCL by the ALK01 protocol showed strong reactivity with the 5A4 antibody. Images of these three cases are shown in Fig. 1A. Close examination revealed that, with the ALK01 antibody, only rare dimly positive cells were detected in the duplicate cores of these three cases. Because it was extremely dim and focal, this signal was originally interpreted as nonspecific background staining. Among the 92 cases that were negative with both the ALK01 and 5A4 protocols, no similar background staining was seen. To further evaluate these three ALK01-dimly positive and 5A4-brightly positive cases, whole sections of these cases were stained with both protocols. Because the ALK01 staining protocol was discontinued in our lab, ALK01 staining of the whole tissue sections was performed at an outside lab that is CLIA-certified and performs ALK01 immunohistochemistry for clinical diagnostic use. In two of these three cases, the ALK01-based protocol showed reactivity that was now interpretable as unequivocally positive (Fig. 1B). A whole tissue section of the third case that was negative for ALK expression in the ALK01-stained tissue microarray but positive with the 5A4-protocol showed comparable staining intensity between the two protocols (Fig. 1B). Fluorescence in situ hybridization using break-apart probes detected *ALK* rearrangement in two of these three cases, with the third case lacking sufficient remaining lymphoma cells for evaluation. Overall, these three cases that were initially interpreted as ALK01-negative but were found to be positive when whole tissue sections were stained were likely initially misclassified due to a paucity of lymphoma cells on the TMA core tissue. Overall, in this cohort, there was 100% concordance between the ALK01 protocol and 5A4 protocol results, with 28 cases positive with both protocols and 95 cases negative with both protocols.

**Fig. 2:**
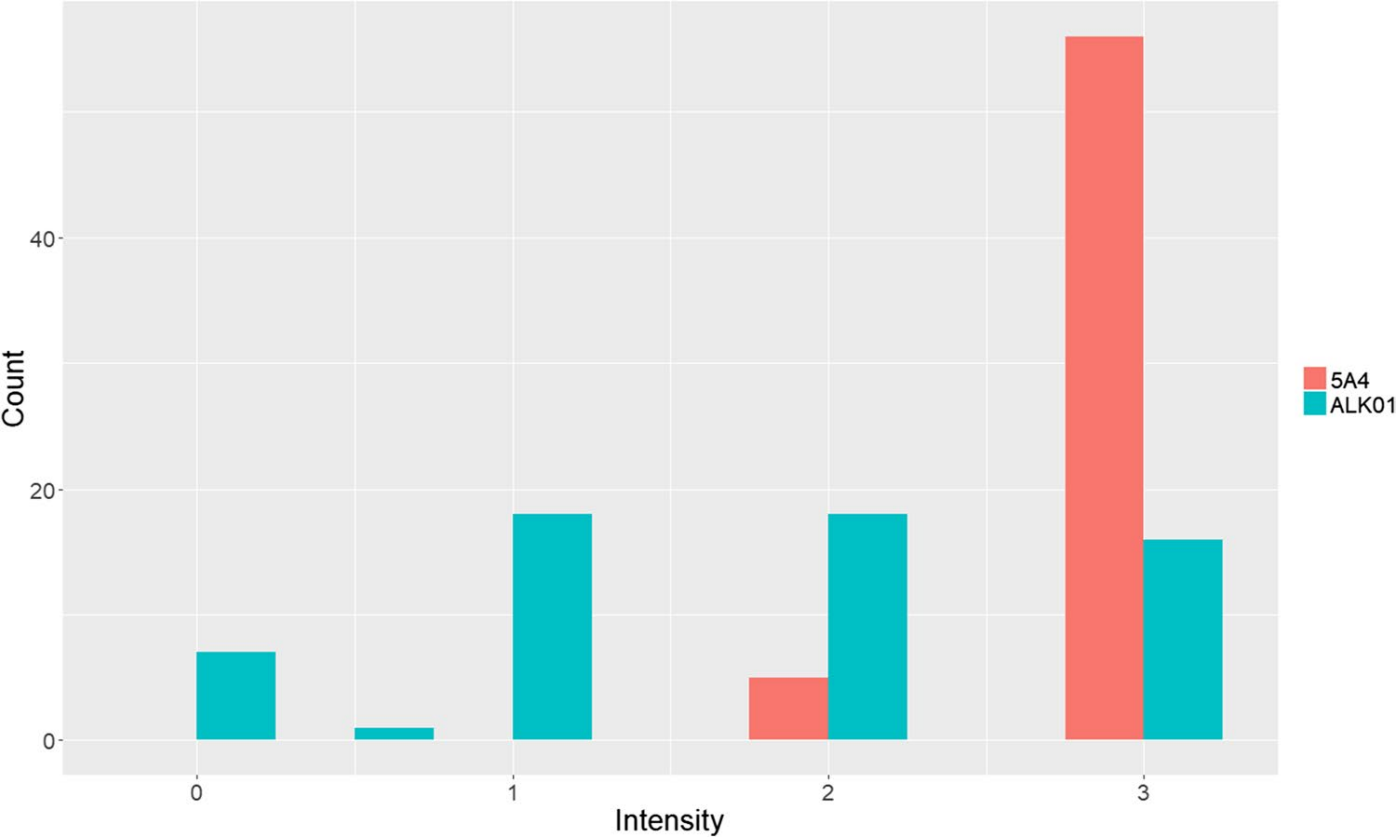
Comparison of the intensity of a stain with the ALK01 and 5A4 protocols. Intensity of the stains was assessed by one of the authors (SFP) on a scale of 0, 1 + , 2 + , and 3 + . The 5A4 protocol tended to show a brighter intensity of signal

Because we noticed that the ALK01 protocol was typically weaker in intensity than the 5A4 protocol, we semi-quantitatively compared the intensity of the ALK signal generated by the two protocols. As shown in Fig. 2, the intensity of our 5A4-based protocol was generally higher than with the ALK01-based protocol. The cellular distribution of the signal was similar with both antibodies in each case.

This study demonstrates that the 5A4 staining protocol initially validated for use in our laboratory for non-hematolymphoid tumors shows equivalent sensitivity and specificity for the detection of ALK-positive ALCL compared with our ALK01 protocol. Based on this study, our laboratory transitioned to using the 5A4-based protocol described here for both hematolymphoid and non-hematolymphoid neoplasms. Recently, another group published their results comparing the sensitivity of the ALK01 clone with a protocol using the D5F3 clone and found greater stain intensity and proportion staining with the D5F3 protocol [14]. Our findings and those of Martin and colleagues may be useful for other laboratories that may also be offering two separate assays for the detection of ALK-positive tumors in hematopoietic and non-hematopoietic tumor types.

## Data Availability

The datasets generated during and/or analyzed during the current study are available from the corresponding author upon request.
